# Mind the Gap: Differential Mental Health Demands in a Brazilian University Campus

**DOI:** 10.1192/j.eurpsy.2026.11304

**Published:** 2026-07-31

**Authors:** C. R. Montalvão, M. Porcu, R. M. Sorgi, M. L. G. Burin, G. C. S. Takizawa

**Affiliations:** UEM, Maringá, Brazil

## Abstract

**Introduction:**

The mental health of university students has become a growing concern in various academic contexts. Studies indicate high prevalences of anxiety and depressive symptoms among students, with significant repercussions on academic performance, interpersonal relationships, and student retention.

**Objectives:**

Describe the profile of demand for psychiatric services on the campus of a Brazilian public institution. Highlighting the distribution by academic centers and discussing possible factors associated with the high prevalence of mental disorders reported in specific courses/fields.

**Methods:**

We analyzed the responses from 176students who received assistance from the university’s psychiatry service during the period from june to august 2025. Data were collected using an institutional form, which included information about the academic center of origin. Responses were systematized and presented in descriptive charts.

**Results:**

The analysis revealed that the largest number of students seeking care belonged to the Center for Human Sciences, Literature, and Arts (37.5%), followed by the Center for Applied Social Sciences (14.2%), Center for Health Sciences (11.9%), Center for Biological Sciences (11.4%), Center for Exact Sciences (9.1%), and Center for Technology (9.1%). The Center for Agrarian Sciences registered the lowest number of attendees.The predominance of students from Humanities, Literature, and Arts, as well as Applied Social Sciences, may be related to multifactorial aspects. These include a greater predisposition to recognize and seek psychological help, curricular characteristics involving a high theoretical load and tight deadlines, in addition to social and cultural contexts that may foster emotional vulnerability. In health courses, such as Medicine, Nursing, and Psychology, the high demand may reflect the impact of early and intense contact with human suffering, as well as the pressure for high academic performance and competitiveness. In the Exact Sciences and Technology areas, although witha lower percentage, a significant demand was also observed, possibly associated with academic stress, social isolation, and activity overload.

**Image 1:**
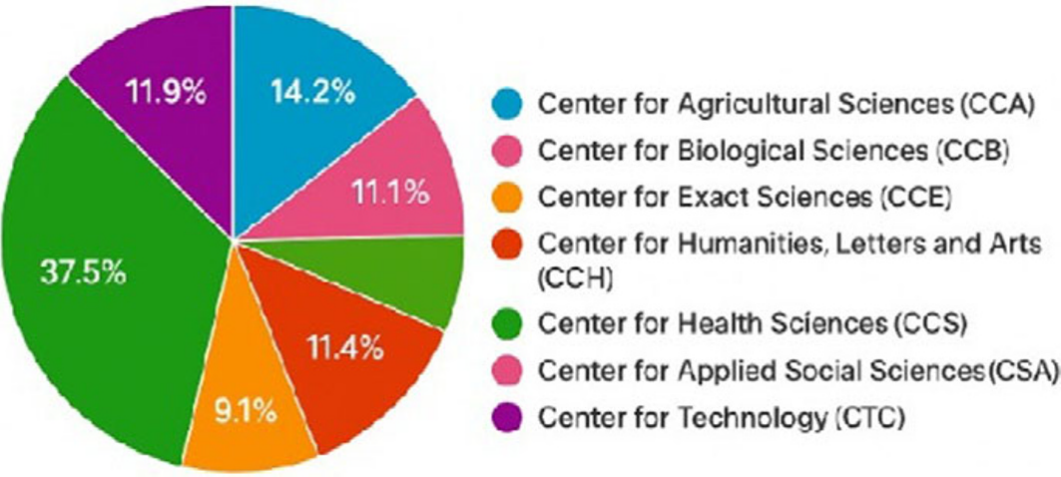
Image 1: Long description.

**Conclusions:**

The findings reinforce the need to strengthen institutional mental health policies, with prevention strategies, integrated multidisciplinary support, and reduction of stigma associated with psychiatric care. Furthermore, they suggest that specific interventions, adapted to the characteristics of each academic center, may be more effective in promoting student mental health.

**Disclosure of Interest:**

None Declared

## Long descriptions

### Image 1: Long description

The pie chart is divided into seven colored segments with the following values clockwise from the top.

* Light blue segment at 14.2 percent.

* Pink segment at 11.1 percent.

* Green segment at 4.8 percent.

* Red segment at 11.4 percent.

* Orange segment at 9.1 percent.

* Large dark green segment at 37.5 percent.

* Purple segment at 11.9 percent.

A legend to the right identifies the centers.

* Light blue represents Center for Agricultural Sciences C C A.

* Pink represents Center for Biological Sciences C C B.

* Orange represents Center for Exact Sciences C C E.

* Red represents Center for Humanities, Letters and Arts C C H.

* Dark green represents Center for Health Sciences C C S.

* A second pink dot represents Center for Applied Social Sciences C S A.

* Purple represents Center for Technology C T C.

Navigate back to Image 1:.

